# Serological Evaluation of Immunity to the Varicella-Zoster Virus Based on a Novel Competitive Enzyme-Linked Immunosorbent Assay

**DOI:** 10.1038/srep20577

**Published:** 2016-02-08

**Authors:** Jian Liu, Xiangzhong Ye, Jizong Jia, Rui Zhu, Lina Wang, Chunye Chen, Lianwei Yang, Yongmei Wang, Wei Wang, Jianghui Ye, Yimin Li, Hua Zhu, Qinjian Zhao, Tong Cheng, Ningshao Xia

**Affiliations:** 1 State Key Laboratory of Molecular Vaccinology and Molecular Diagnostics, National Institute of Diagnostics and Vaccine Development in Infectious Diseases, School of Life Sciences, Xiamen University, Xiamen, 361102, China; 2 Beijing Wantai Biological Pharmacy Enterprise, Beijing, 102206, China; 3 Department of Microbiology and Molecular Genetics, New Jersey Medical School, Rutgers University, 225 Warren Street, Newark, NJ 070101, USA

## Abstract

Varicella-zoster virus (VZV) is a highly contagious agent of varicella and herpes zoster. Varicella can be lethal to immunocompromised patients, babies, HIV patients and other adults with impaired immunity. Serological evaluation of immunity to VZV will help determine which individuals are susceptible and evaluate vaccine effectiveness. A collection of 110 monoclonal antibodies (mAbs) were obtained by immunization of mice with membrane proteins or cell-free virus. The mAbs were well characterized, and a competitive sandwich ELISA (capture mAb: 8H6; labelling mAb: 1B11) was established to determine neutralizing antibodies in human serum with reference to the FAMA test. A total of 920 human sera were evaluated. The competitive sandwich ELISA showed a sensitivity of 95.6%, specificity of 99.77% and coincidence of 97.61% compared with the fluorescent-antibody-to-membrane-antigen (FAMA) test. The capture mAb 8H6 was characterized as a specific mAb for VZV ORF9, a membrane-associated tegument protein that interacts with glycoprotein E (gE), glycoprotein B (gB) and glycoprotein C (gC). The labelling mAb 1B11 was characterized as a complement-dependent neutralizing mAb specific for the immune-dominant epitope located on gE, not on other VZV glycoproteins. The established competitive sandwich ELISA could be used as a rapid and high-throughput method for evaluating immunity to VZV.

Varicella-zoster virus (VZV) is a highly contagious agent belonging to the subfamily *Alphaherpesvirinae*. Primary infection of VZV causes varicella, while reactivation of latent virus from sensory ganglia results in herpes zoster (HZ)^1^. Postherpetic neuralgia (PHN) is a severe sequela of HZ and the most common, especially in the aged^2^. VZV infections are more severe in immunocompromised patients and may come with severe complications, such as encephalomyelitis^3^, varicella gangrenosa^4^, myelitis^5^. Effective vaccines have been developed to prevent VZV infection^6^,^7^, however, they are limited by the scope of vaccination and in some cases vaccine efficacy^8^, and VZV remains an important pathogen. In particular, laboratory determination of the status of immunity to VZV has been recommended for immunocompromised patients, pregnant women and health workers after exposure to VZV^9^.

VZV-specific antibodies in serum could be used as a reliable indicator for serological confirmation of VZV-specific immunity^10^. A highly sensitive and specific serological test is important for obtaining herd immunity against VZV. Several methods have been developed to measure serum VZV-specific immunoglobulin G (IgG) antibodies, including the fluorescent-antibody-to-membrane-antigen (FAMA) test^11^,^12^, an enzyme-linked immunosorbent assay (ELISA) based on total VZV antigen or purified glycoproteins (gps)^13^,^14^,^15^ and many other methods^10^,^16^,^17^,^18^,^19^,^20^,^21^. ELISA based on glycoproteins (gpELISA) has been used to detect seroconversion in vaccinated populations^13^,^15^,^18^. The FAMA test is regarded as the “gold standard” indication for immunity to varicella^8^,^11^. The titre of FAMA test is highly correlated with the level of neutralizing antibody and susceptibility to varicella^22^,^23^,^24^. Furthermore, the result of the FAMA test is officially approved, and it is recommended that new assays use the FAMA test as reference.

The detection mode of the FAMA test is based on immunofluorescence of membrane proteins on virus-infected cells. It is laborious and low-throughput. While detected by gpELISA, Serum with a titre ≥ 5 gpELISA units/mL has been used to indicate protective antibodies^13^. However, the gps antigen varies between antigen lots, and internal serum panels are needed for antigen qualification. Competitive ELISA has been used to detect serum neutralizing antibodies against porcine circovirus and rabies virus^25^,^26^,^27^, but not VZV. The objective of the present study was to develop a competitive ELISA-based protective antibody detection method for serological testing of VZV-specific immunity. Cell-free virus, purified recombinant glycoproteins and VZV-gps were used to immunize BALB/c mice, and a collection of 110 mAbs were obtained. The mAbs were well characterized by neutralization assay, blocking ELISA, and capture ELISA coupled with quantitative real-time PCR. Finally, a pair of mAbs were selected to establish a competitive ELISA, which was highly coincident with the FAMA test in 920 human sera detection. Capture mAb (8H6) was identified as an mAb specific for ORF9, further characterizing the membrane-associated tegument protein that interacts with gE, gB and gC. Labelling mAb (1B11) was characterized as a neutralizing mAb specific for the immune-dominant epitope located on gE. The established competitive ELISA could be used as a rapid and high-throughput method to determine the status of immunity to VZV.

## Results

### Preparation of recombinant VZV membrane proteins and mAbs

The fragment of gE (1–537 aa) was expressed using the baculovirus expression system, and purified as previously reported^28^. The other membrane proteins were expressed using the *E. coli* expression system. The fragments of gH (518–804 aa) and gL (23–160 aa) were expressed as inclusion bodies, which were solubilized in 8 M urea, purified with Ni-NTA and refolded. The GST-fused fragment of gK (22–114 aa) and the fragment of gB (23–382 aa) were expressed as inclusion bodies and purified by electroelution. The fragments of gI (21–274 aa), gM (2–35 aa) and gC (18–407 aa) were expressed as GST fusion proteins and purified with glutathione Sepharose 4B. The GST tag fused to gC was removed with PreScission protease (PPase). The fragment of gN (21–49 aa) was fused to a linker (GSGGSG) and repeated four times; the new construct was named gN-L. gN-L was fused to a His tag and purified with Ni-NTA. Purified membrane proteins, VZV gps and cell-free virus were used to immunize mice, and a collection of 110 mAbs were obtained (Table 1).

### Establishment of a competitive sandwich ELISA to detect serum neutralizing antibody

**Figure 1:**
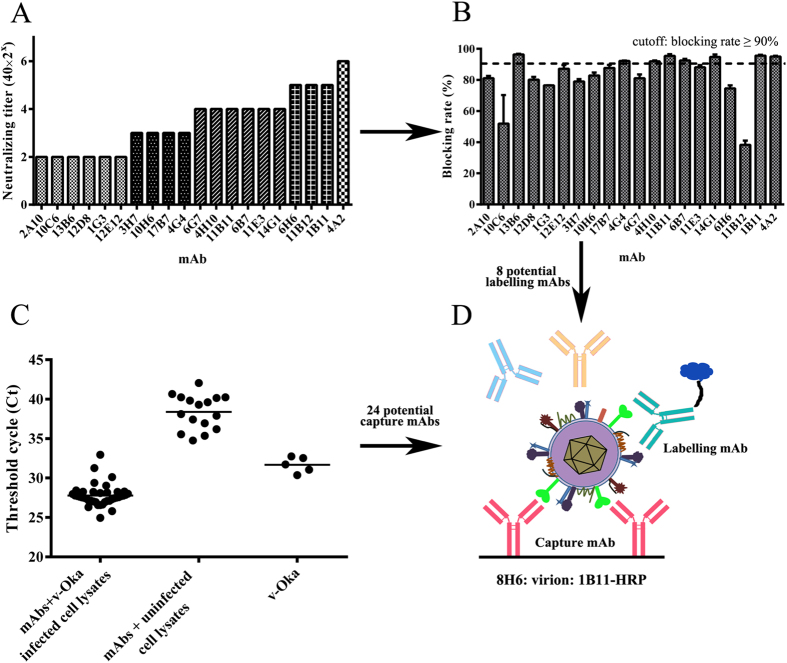
Establishment of a competitive sandwich ELISA to detect serum neutralizing antibody. (**A**) 110 mAbs were obtained by immunization of mice with cell-free virus, purified VZV-gps and recombinant membrane proteins. All the mAbs were screened with an Elispot-based neutralization assay, and 20 complement-dependent neutralizing mAbs were obtained. (**B**) Blocking ELISA was performed with positive human serum. 8 neutralizing mAbs with a blocking rate greater than 90% were selected as potential labelling mAbs. (**C**) The ability of mAbs to capture VZV virions was evaluated by combining capture ELISA with quantitative real-time PCR. 24 mAbs in group I with threshold cycle (Ct) lower than average (27.78) were selected as potential capture mAbs. (**D**) Pairing experiment. Two rounds of pairing experiments were conducted to select a pair of mAbs for serum evaluation. In the first round of the pairing experiment, 192 combinations (24 × 8) were tested with one positive serum (FAMA titre = 1:16) and one negative serum (FAMA titre < 1:2), and 13 combinations were selected; in the second round of the pairing experiment, a panel of 20 human sera (12 positive sera (FAMA titre ≥ 1:8) and 8 negative sera (FAMA titre < 1:2)) were used for a correlation experiment. A pair of mAbs (8H6/1B11-HRP) were selected to established the competitive sandwich ELISA, which had a 100% coincidence rate with the FAMA test in the second pairing experiment.

Twenty mAbs were identified as complement-dependent neutralizing mAbs by the Elispot-based neutralization assay (Fig. 1A). Blocking ELISA was used to identify neutralizing antibodies responding to the immunodominant epitope. Eight neutralizing mAbs with a blocking rate greater than 90% were selected as potential labelling mAbs (Fig. 1B).

Capture ELISA coupled with quantitative real-time PCR was used to evaluate the ability of mAbs to capture VZV virions. Forty mAbs were coated on ELISA plates and incubated with cell-free virus (group I). Uninfected ARPE-19 cell lysates with mAbs coated (group II) and cell-free virus without mAbs coated (group III) were set as control. From the results of quantitative real-time PCR (Fig. 1C), the mean threshold cycle (Ct) of group I was 27.78, while those of group II and group III were 38.39 and 31.68. Twenty-four mAbs in group I with Ct lower than 27.78 were selected as potential capture mAbs.

Serum with FAMA titre ≥ 1:2 was considered positive^10^, while serum with FAMA titre ≥ 1:8 (four-fold or greater increase) was considered to contain protective (neutralizing) antibodies^13^. A blocking rate of competitive sandwich ELISA greater than 50% was considered positive. Twenty-four capture mAbs and 8 labelling mAbs were used to perform a pairing experiment. One positive serum (FAMA titre = 1:16) and one negative serum (FAMA titre < 1:2) were used in first pairing experiment. In second pairing experiment, thirteen pairs of mAbs were selected for a correlation experiment using a panel of serum containing 12 positive sera (FAMA titre ≥ 1:8) and 8 negative sera (FAMA titre < 1:2). A competitive sandwich ELISA based on a pair of mAbs (8H6/1B11-HRP) was highly coincident with that of the FAMA test (coincidence rate = 100%).

### Evaluation of human serum using the competitive sandwich ELISA in comparison to the FAMA test

Nine hundred twenty human serum samples were evaluated by the competitive sandwich ELISA and FAMA test. The serum samples were divided into two groups, group I containing 820 sera collected from 9-month-old to 12-year-old children, group II containing 100 sera collected from adults. The FAMA titre of 380 sera was greater than 1:8 in group I, while 97 sera were greater than 1:8 in group II. All serum samples were tested by the established competitive sandwich ELISA. In group I, there were 361 serum samples with a blocking rate greater than 50%, while the blocking rate of the other 459 serum sample was less than 50% (Table 2). Compared with the FAMA test, the competitive sandwich ELISA showed a sensitivity of 94.7%, a specificity of 99.77% and a coincidence rate of 97.19% in children’s serum (Table 3). In group II, there were 97 serum samples with a blocking rate greater than 50%, while that of the other 3 serum samples was less than 50% (Table 2). The competitive sandwich ELISA showed a sensitivity of 98.97%, a specificity of 100% and a coincidence rate of 99.00% in adult serum (Table 3). The competitive sandwich ELISA was highly correlated to the FAMA test in testing for VZV-specific immunity.

### Identification of the labelling mAb 1B11 and capture mAb 8H6

**Figure 2:**
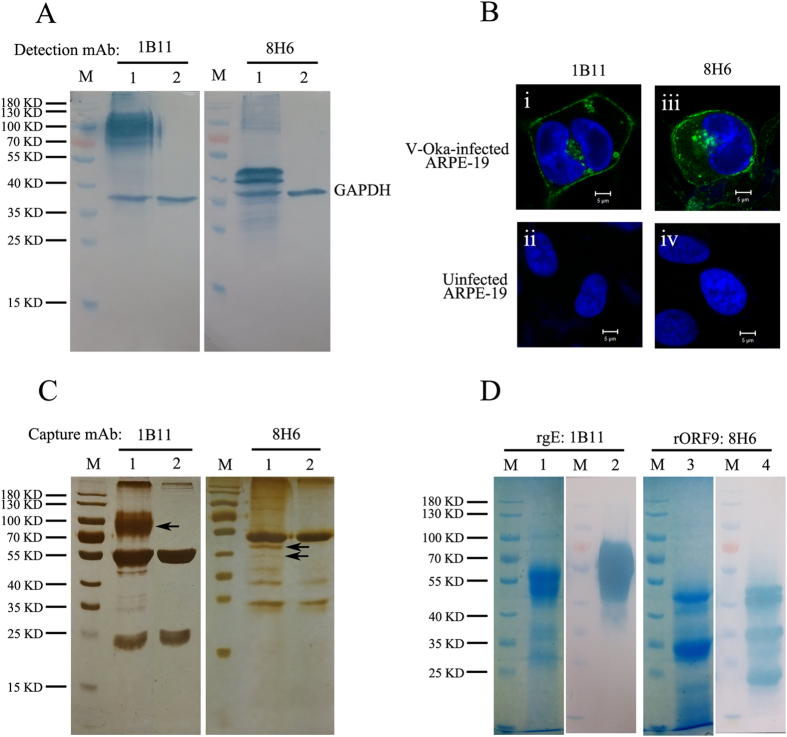
Identification of labelling mAb 1B11 and capture mAb 8H6. (**A**) Immunoblot of v-Oka-infected ARPE-19 cell lysates (lane 1) and uninfected ARPE-19 cell lysates (lane 2) with labelling mAb 1B11 and capture mAb 8H6. Both 1B11 and 8H6 were specific to a component of VZV. The molecular weights of the proteins recognized by 1B11 ranged from 70 to 110 kD, while 8H6 recognized two bands ranging from 38 to 50 kD. GAPDH (Proteintech, Wuhan, China) was used as the internal reference. (**B**) Confocal immunofluorescence microscopy was performed to verify the reaction of 1B11 and 8H6 with v-Oka-infected ARPE-19 cells. The protein that 1B11 was specific for was present on the plasma membrane and in some compartments of the cytoplasm (i). The protein that 8H6 was specific for was present on the plasma membrane and in the cytoplasm (iii). Both mAbs did not react with uninfected ARPE-19 cells (ii & iv). Nuclei were stained with DAPI. The image magnification was × 630. (**C**) 1B11 and 8H6 were incubated with v-Oka-infected (lane 1) and uninfected ARPE-19 cell lysates (lane 2) and immobilized with protein A beads. The bound proteins were separated by SDS-PAGE and revealed by silver staining. Compared with uninfected cell lysates, the specific proteins (indicated with black arrows) with the right molecular weight were excised and identified by mass spectrometry.(**D**) Recombinant gE and ORF9 were purified and immunoblotted with 1B11 and 8H6. 1B11 reacted with rgE, while 8H6 reacted with rORF9.

Both labelling mAb 1B11 and capture mAb 8H6 reacted with v-Oka-infected cell lysates (Fig. 2A, lane 1), while no band was detected in uninfected ARPE-19 cell lysates (Fig. 2A, lane 2) in the western blot. The protein 1B11 recognized ranges from 70 to 110 kDa, while the protein 8H6 recognized ranges from 38 to 50 kDa. GAPDH (Proteintech, Wuhan, China) was used as the internal reference in the experiment. In the immunofluorescence (IF) assay, a high fluorescence signal was detected on the virus-infected plasma membranes and some compartments in the cytoplasm after incubation with 1B11 (i). When v-Oka-infected ARPE-19 cells were incubated with 8H6, a fluorescence signal was detected on the plasma membrane and in the cytoplasm; (iii) No signal could be detected in uninfected cells (ii,iv). (Figure 2B)

To identify which proteins 1B11 and 8H6 recognized, immunoprecipitation (IP) coupled with mass spectrometry was performed. Proteins captured by the two mAbs were separated by SDS-PAGE and revealed by silver staining. Compared with uninfected cell lysates (negative control, Fig. 2C, lane 2), specific proteins with the right molecular weights (MW) (Fig. 2C, lane 1, indicated by black arrows) were excised, and their sequences were identified by mass spectrometry. The band pulled down by 1B11 was gE, while both bands pulled down by 8H6 were ORF9. Recombinant VZV gE and ORF9 were expressed, purified and immunoblotted with the two mAbs. Western blotting demonstrated that the labelling antibody 1B11 reacted with rgE (Fig. 2D, lanes 1 and 2), while the capture antibody 8H6 reacted with rORF9 (Fig. 2D, lanes 3 and 4). Based on Western blotting, the two antibodies were expected to recognize the linear epitope located on rgE or rORF9. Furthermore, 15-mer peptides with 5 aa overlapping, covering the entire sequence of gE and ORF9, were used to react with the labelling 1B11 antibody and the capture 8H6 antibody to identify the peptides that the antibodies bound to. However, 1B11 and 8H6 failed to react with any 15-mer peptides. Subsequently, a truncated antigen expression strategy was used to identify the approximate regions 1B11 and 8H6 binding to gE or ORF9. The labelling mAb 1B11 bound to the gE residues 140–330 and the capture mAb 8H6 bound to the ORF9 residues 81–135. The gE residues 140–330 recognized by 1B11 partly covered the gE residues 1–134 and 101–161 that have previously been identified as immunodominant regions in the published literature^29^.

### Characterization of labelling mAb 1B11 and VZV ORF9

**Figure 3:**
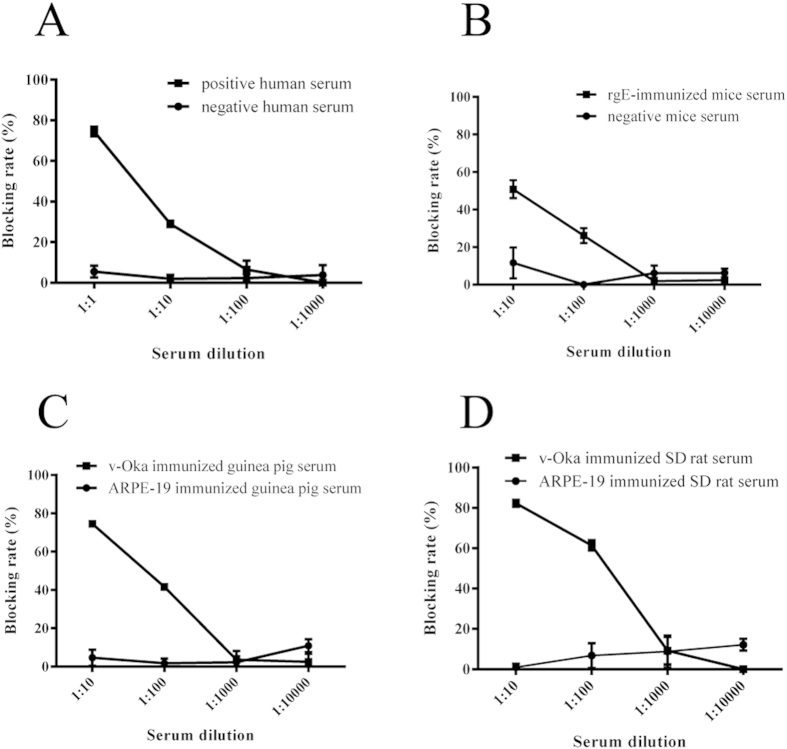
The epitope that 1B11 is specific for is immunodominant in human and animals. Positive and negative sera from human (natural infection), mouse (immunized with rgE), guinea pig (immunized with v-Oka) and SD rat (immunized with v-Oka) were used in the blocking ELISA. Positive sera from human, mouse, guinea pig and SD rat effectively block 1B11 binding to rgE. The epitope that 1B11 was specific for was immunodominant, and the established sandwich ELISA could also be used to detect mAbs in these animals.

Positive and negative sera from human, mouse (immunized with rgE), guinea pig (immunized with v-Oka or ARPE-19) and SD rat (immunized with v-Oka or ARPE-19) were used to block labelling mAb 1B11 from binding to rgE. The results showed that the epitope that 1B11 was specific for was immunodominant in human (Fig. 3A), mouse (Fig. 3B), guinea pig (Fig. 3C), and SD rat (Fig. 3D). Thus, the constructed competitive sandwich ELISA could also be applied to evaluate antibodies in small animals’ sera.

**Figure 4:**
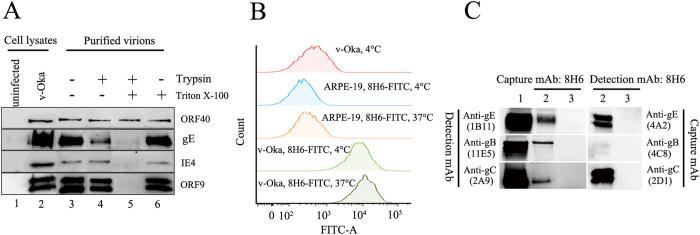
ORF9 is a membrane-associated tegument protein and interacts with gE, gB and gC. (**A**) ARPE-19 cells were infected with v-Oka, and cell-free viruses were purified by sucrose density gradient centrifugation. Purified virions were dialyzed to PBS and then treated with trypsin (0.2 mg/ml) in either the presence or absence of 1% Triton X-100 for 25 min at room temperature. Equivalent amounts of virion lysates were analysed for the indicated viral proteins in Western blotting. (**B**) v-Oka-infected ARPE-19 and uninfected ARPE-19 cells were incubated with 8H6-FITC at 37 °C or 4 °C for 30 min. v-Oka-infected ARPE-19 cells incubated with PBS at 4 °C for 30 min were used as the controls. All the samples (approximately 5,000 cells per test) were analysed using a FACSAria III cell sorter (BD Biosciences, San Jose, California, USA), and v-Oka-infected ARPE-19 cells incubated with 8H6-FITC showed a significant shift to higher fluorescence intensities. (**C**) Coimmunoprecipitation (Co-IP) of ORF9 with VZV membrane proteins *in vitro*. Uninfected and v-Oka-infected ARPE-19 cells were harvested in 1% Triton X-100 lysis buffer. Cell lysates (precleared with protein A beads) were incubated with 8H6 and then incubated with protein A beads. After fully washing with PBST (0.05% Tween 20 in PBS), the bound proteins were detected with VZV membrane protein mAbs by Western blot. Compared with uninfected cell lysates (lane 3), specific bands were detected by gE mAb (1B11), gC mAb (2A9) and gB mAb (11E5). The reaction of these mAbs with v-Oka-infected cell lysate was verified (lane 1). Furthermore, gE mAb (4A2), gC mAb (2D1) and gB mAb (4C8) were used to pull down ORF9, and 8H6 was used detection in the Western blot. In contrast to the uninfected cell lysate (lane 5), ORF9 was pulled down by these mAbs (lane 4).

VZV ORF9 has been identified as a tegument protein by immuno-gold staining^30^. However, the mAb specific for ORF9 was used as a capture mAb and paired with a mAb specific for gE in serological detection. In a previous study, cell-free virus was purified by sucrose density gradient centrifugation^31^. To verify the location of ORF9 within virus particles, purified cell-free virus was treated with trypsin in either the absence or presence of Triton X-100. gE was sensitive to trypsin in both the absence (Fig. 4A, lane 4) and the presence (Fig. 4A, lane 5) of Triton X-100; the major capsid protein ORF40 was resistant to trypsin even in the presence of Triton X-100 (Fig. 4A, lane 5). In contrast, ORF9 and tegument protein IE4 were sensitive to trypsin only in the presence of Triton X-100 (Fig. 4A, lane 4 and 5). These results verified that ORF9 was present in the virion tegument.

ORF9 demonstrated plasma membrane localization (Fig. 2B iii), and membrane-associated ORF9 was further examined using flow cytometry. After incubating with 8H6-FITC, v-Oka-infected ARPE-19 cells showed a significant shift to higher fluorescence intensities (Fig. 4B), verifying the ability of ORF9 to associate with the cellular membrane.

VZV ORF9 interacts with gE in VZV-infected melanoma cell^30^. Capture mAb 8H6 was used to pull down potential protein complex in v-Oka-infected ARPE-19 cell lysates, and the interaction was probed with mAbs specific for membrane proteins in western blotting. ORF9 interacted with gE, gB and gC (Fig. 4C, lane 2 and 3). mAbs specific for gE (4A2), gB (4C8) and gC (2D1) were used as capture mAbs and the complex was probed with 8H6. The results verified the interaction between gE, gB, gC and ORF9 (Fig. 4C, lane 4 and 5).

**Figure 5:**
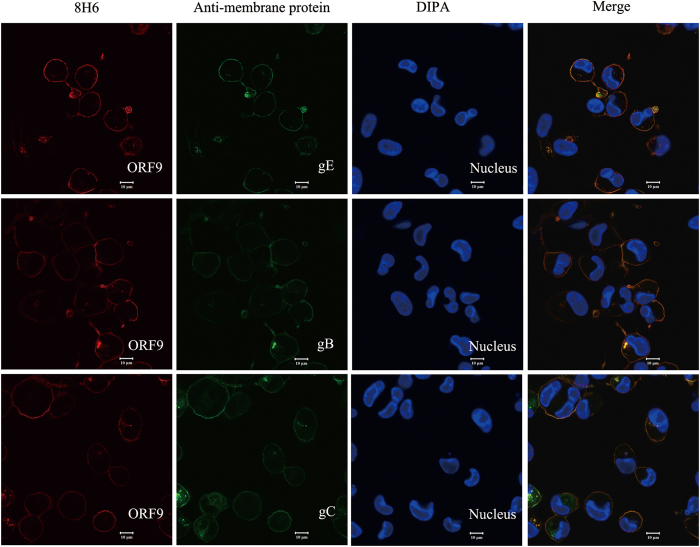
Localization of ORF9 and gE, gB, gC in v-Oka-infected ARPE-19 cells. v-Oka-infected ARPE-19 cells were fixed, permeated, blocked and incubated with 8H6, followed by incubation with GAM-TRITC (red). Subsequently, cells were incubated with FITC labelled mAbs specific for gE, gB and gC respectively (green). The nucleus was stained with DAPI (blue). The respective staining panels were merged, and the co-localization between ORF9 and gE, gB, gC was analysed.

Confocal microscopy was performed to verify the interaction between ORF9 and membrane proteins using ZEISS LSM780 (ZEISS, Oberkochen, Germany). v-Oka-infected ARPE-19 cells were incubated with 8H6 and GAM-TRITC (Sigma-Aldrich, St. Louis, Missouri, USA) and subsequently incubated with FITC labelled mAbs 4A2, 4C8 and 2A9, respectively. The nucleus was stained with DAPI. The samples were analysed using a dual-immunofluorescent microscopy for the co-localization of ORF9 and membrane proteins. Confocal imaging confirmed the co-localization between ORF9 and gE, gB, gC (Fig. 5).

## Discussion

VZV is a highly contagious virus that can cause varicella and herpes zoster. More than 90% of susceptibles could be infected after a household exposure to the virus^32^. In immunocompromised persons and adults with varicella, the symptoms could be much severe. Maternal varicella during gestation may cause congenital varicella syndrome^33^. Varicella may also couple with bacterial infection and cause severe complications. Prevention and control of VZV infection is particularly important. VZV-specific antibodies are thought to be key to prevent varicella. Serological evaluation of VZV-specific antibodies is the most important indicator to determine susceptibility and the effectiveness of a varicella vaccine. Furthermore, determination of baseline of VZV-specific antibodies in a population can help establish a proper immunization programme.

In previous reports, VZV-infected whole cell lysates, total VZV gps or gpI (gE), gpII (gB), and gpIII (gH) purified from VZV-infected cell lysates have been used as coating proteins to establish indirect ELISA to evaluate VZV-specific IgG^14^,^15^,^34^. However, VZV whole-cell ELISA was not very sensitive, and total VZV gps based ELISA has been thought to yield false-positive results and overestimate of the rate of seroconversion^22^,^35^. Blocking ELISA has been used to measure levels of neutralization-related antibodies to many other viruses^25^,^26^. In the detection mode, virus-specific antibody is coated onto the ELISA plate to capture virus or recombinant virus protein; labelling neutralizing mAbs are used to block serum antibodies; and the blocking rate indicates the presence of neutralizing antibody in the serum. The employment of HRP-labelled mAbs against the glycoprotein of VZV and elimination of the use of a secondary antibody may improve the specificity of the current ELISAs, which frequently need to subtract non-specific reactions caused by uninfected cell components. This study adopted the FAMA test as reference to establish a blocking ELISA to evaluate the levels of neutralizing mAbs in human serum.

VZV expresses at least nine membrane proteins, many of which are highly immunogenic^34^. Several neutralizing epitopes have been reported to locate on gE, gB and gH, inducing complement-dependent or complement-independent neutralizing antibodies^36^,^37^,^38^,^39^,^40^. In this study, the baculovirus or *E. coli* expression system was used to express the extracellular domains of VZV membrane proteins. Considering gE is an essential and most abundant membrane protein, the baculovirus expression system was used to express the extracellular domain of gE (rgE) similar to the published article^28^,^41^. The results showed that the rgE antigen could elicit plenty of antibodies recognizing linear or conformational epitopes locate on native gE, and neutralizing mAbs used in this study were obtained from rgE immunized mice. The *E. coli* expression system was also used to express extracellular domains of other VZV membrane proteins due to its high efficiency. The results showed that fragments of rgI, rgC, rgM and rgN were soluble, while fragments of rgB, rgH, rgK and rgL were expressed in the form of inclusion bodies. Whether these antigens expressed by the *E. coli* expression system maintain important B-cell or T-cell epitopes requires further evaluation. However, these antigens could be used to immunize mice to prepare the candidate mAbs.

A total of 110 mAbs specific for VZV proteins were obtained by immunizing mice with cell-free virus or the purified membrane proteins. In this study, neutralization test, blocking ELISA, capture ELISA, quantitative real-time PCR and pairing experiment were performed and a pair of mAbs was selected to establish a competitive ELISA. The established competitive ELISA and the FAMA test were used to detect 920 human sera in parallel. The competitive ELISA had a sensitivity of 95.6%, specificity of 99.77% and coincidence of 97.61% with the FAMA test.

The pair of mAbs were shown to be specific for VZV proteins in Western blotting and IF assays. 1B11 was identified as a gE mAb bound to gE residues 140–330, and 8H6 was identified as a ORF9 mAb bound to ORF9 residues 81–135. VZV gE is the major and most immunogenic membrane protein; some gE-specific mAbs have been obtained, and some epitopes had been identified^29^,^41^,^42^. The residues 140–330 of gE recognized by 1B11 partly cover the residues 1–134 and 101–161 of gE that were previously identified as immunodominant regions in published literature^29^. It was an unexpected finding that a tegument protein mAb could pair with a mAb specific for a membrane protein in a sandwich ELISA. In this study, cell-free virus was purified by sucrose density gradient centrifugation and treated with trypsin in either the absence or presence of Triton X-100. The results showed that ORF9 was sensitive to trypsin in the presence of Triton X-100, which verified that ORF9 is incorporated into VZV virions and present in the virion tegument^30^. One unexpected finding was that ORF9 is located on the plasma membrane, which was observed by confocal microscopy and verified using flow cytometry. HSV-1 VP22, the homologue of ORF9, was also reported to be a membrane-associated protein^43^,^44^. As previously reported, ORF9 interacts with gE in VZV-infected melanoma cells. In this study, coimmunoprecipitation was performed using v-Oka-infected ARPE-19 cell lysates. ORF9 interacts with not only gE but also gB and gC. Interaction between ORF9 and gB or gC has not been reported previously. Furthermore, using a double-staining procedure, the co-localization between ORF9 and gE, gB, gC was observed using confocal microscopy. The interaction between ORF9 and membrane proteins might bring ORF9 to the v-Oka-infected cells and virions membrane, which was indicated by the IF assay (Fig. 5) and the virion capture experiment (Fig. 1C). Based on these results, the detection format of the established competitive ELISA might include capture of virions and ORF9-gE complex by 8H6.

VZV-specific antibodies are currently thought to play an important role in preventing primary VZV infection, and T-cell responses are thought to have an important role in recovering from varicella and reactivating of latent virus^8^,^45^. Although VZV infection or vaccination could elicit both humoral and cellular immunity, there is still no strong evidence to support that the level of VZV-specific antibody will always be correlated with T cell response. In this study, the established competitive ELISA has been validated for serological evaluation, but whether the data of the assay could be used for evaluate T-cell response to VZV still needs further research.

In summary, the established competitive ELISA performed well in human serum detection. Furthermore, positive serum from rgE-immunized mice and v-Oka-immunized guinea pigs and SD rats blocked 1B11 binding to rgE. The epitope that 1B11 is specific for is immunodominant in these three small animals and humans. The established competitive sandwich ELISA could also be used to evaluate neutralizing antibodies in both human and the three animal sera.

## Methods

### Cell and virus

Human acute retinal pigment epithelial cells (ARPE-19, purchased from ATCC, Manassas, Virginia, USA) and human foetal lung fibroblast cells (MRC-5, purchased from ATCC, Manassas, Virginia, USA) were routinely maintained in Dulbecco’s modified Eagle’s medium (DMEM) with 10% foetal bovine serum (FBS, PAA, Hyclone, Logan, Utah, USA). *Spodoptera frugiperda* (Sf21) cells (Invitrogen, Carlsbad, CA, USA) were routinely maintained in CCM_3_ medium (Hyclone, Logan, Utah, USA) with 2% FBS. SP2/0 cells were grown in RPMI 1640 medium (Invitrogen, Carlsbad, CA, USA) with 10% FBS.

The vaccine Oka strain (v-Oka) was maintained in ARPE-19 or MRC-5 cells. v-Oka-infected cells with 80% cytopathic effect (CPE) were scraped into the VZV protection buffer (9% sucrose, 25 mM histidine, 150 mM NaCl, pH 7.35) and stored at −80 °C. Aliquots were thawed and centrifuged at low speed (400 × *g*, 15 min) to obtain cell-free virus. Baculovirus (*Autographa californica* nuclear polyhedrosis virus, AcMNPV) was purchased from Clontech (Mountain View, California, USA).

### Expression and purification of VZV proteins

VZV-gps were prepared and purified from v-Oka-infected MRC-5 cells as described in previous work^15^. Control antigen from uninfected MRC-5 cells were prepared using the same method. In brief, VZV gps from v-Oka-infected MRC-5 cells with 80% cytopathic effect were solubilized with 50 mM Tris-HCl, pH 7.5, 2% (v/v) Triton X-100, 4 mM phenylmethyl sulfonyl fluoride (PMSF) and purified using Lectil lectin sepharose (GE Healthcare, Uppsala, Sweden).

A fragment of glycoprotein E (rgE, 1–537aa, GenBank No. AAY57748) fused to a His tag was expressed by following the protocol of the Bac-to-Bac system (Invitrogen, Carlsbad, CA, USA) as previous report^28^,^41^. Other membrane protein fragments and ORF9 were expressed using an *Escherichia coli (E. coli*) expression system. Membrane protein fragment and ORF9 genes were cloned from the genome of v-Oka (GenBank No. DQ008354.1) and into pET-30a (+) (Novagen, Darmstadt, Germany) or pGEX-6p-1 (Amersham Biosciences AB, Uppsala, Sweden). Recombinant proteins were purified by Ni-NTA agarose (Qiagen, Venlo, Netherlands) or glutathione Sepharose 4B (GE Healthcare, Uppsala, Sweden).

### Preparation of mAbs against VZV

Cell-free virus, purified VZV gps and recombinant membrane proteins were used to immunize specific pathogen-free BALB/c mice and boosted at 2-week intervals twice. Spleen cells were fused with sp2/0 (myeloma cells). Hybridoma supernatant was tested by indirect ELISA and positive wells were cloned at least twice. Ascetic fluid produced from a single positive clone was purified by protein A chromatography (GE Healthcare, Uppsala, Sweden). All experiments were performed in accordance with institutionally approved protocols and the School of Life Science Guide for the care and use of laboratory animals, and all experimental protocols were approved by the Xiamen University Laboratory Animal Management Ethics Committee.

### Neutralization assay

The ability of mAbs to neutralize virus was tested using a modified enzyme-linked immunosorbent spot (Elispot) - based neutralization assay^46^. In brief, 100 plaque forming units (PFU) of cell-free virus were incubated with serially diluted mAbs with or without a complement (1:10) for 1 h at 37 °C, and then incubated with ARPE-19 cells with 50% confluence pre-seeded in 24-well plates (NUNC, Roskilde, Denmark) for 1 h at 37 °C. The supernatant was replaced with fresh medium and incubated for three days. An Elispot assay was performed, and spot-forming cells were counted. The dilution of mAb that could neutralize half of the virus was set as the neutralization titre.

### Determination of the ability of mAbs to capture virions by combining capture ELISA and quantitative real-time PCR

Purified mAbs or diluent (blank control) was coated onto 96-well ELISA plates (200 ng/well), after blocking non-binding sites with blocking buffer (0.5% casein, 2% gelatin, 0.1% proclin-300, 10% sucrose). Then, 100 μL cell-free virus or uninfected cell lysate was added to each well. After incubation at 37 °C for 1 hour, the plates were washed ten times with PBS, then 100 μL ddH_2_O was added to each well and incubated at 95 °C for 15 min. Samples collected from the well were used to determine the genome copy number using a fluorogenic polymerase chain reaction assay^47^.

### Blocking ELISA for detection of serum antibodies against membrane proteins

mAbs were covalently conjugated to horseradish peroxidase (HRP) using the periodate oxidation method^48^. The dilution of labelling mAbs to an OD_450_ value between 0.8 and 1.2 was confirmed by direct ELISA. Positive and negative sera with ten-fold serial dilutions were used to block VZV mAb binding to antigen coated on the ELISA plates. Percent inhibition (PI) was calculated using the formula: PI(%) = 100 × [1-(positive serum OD_450_/negative reference serum OD_450_)].

### The FAMA test

The FAMA test has been considered the “gold standard” for measuring immunity to VZV^9^,^10^,^22^,^49^. In short, v-Oka-infected MRC-5 cells were incubated first with diluted human serum and then with anti-human labelled with fluorescein isothiocyanate (GAH-FITC, Abcam, Cambridge, UK), pre-mixed with Evans blue. The result was viewed by a fluorescence microscope IX71 (OLYMPUS, Tokyo, Japan).

### Epitope analysis using pepscan and protein fragments

Pepscan analysis was used to identify the epitope of mAb 1B11 and 8H6. In total, 54 synthetic 15-mer peptides with 5-mer overlaps, covering the 1-537 gE sequence, and 30 synthetic 15-mer peptides with 5-mer overlaps, covering the 1-302 ORF9 sequence, were used to coat the ELISA plates (1 μg/well) and react with mAbs 1B11 and 8H6 by indirect ELISA. Fragments of rgE and ORF9 fused to GST tag were expressed by the *E. coli* expression system and used to react with mAb 1B11 and 8H6 by Western blotting. In this study, eleven gE fragments spanning 1–537 aa, 1–194 aa, 140–330 aa, 271–430 aa, 398–537 aa, 188–330 aa, 214–330 aa, 237–330 aa, 264–330 aa, 188–299 aa, and 140–194 aa and eight ORF9 fragments spanning 1–302 aa, 1–53 aa, 40–96 aa, 82–136 aa, 118–176 aa, 159–215aa, 200–255 aa, and 240–302 aa were expressed.

### Purification and analysis of cell-free virus

The procedure employed in this study to purify cell-free virus was as previously reported^31^. Purified cell-free virus derived from fractions of a sucrose gradient was dialyzed to PBS buffer (pH 7.4) and treated with 0.2 mg/mL trypsin in either the presence or absence of 1% Triton X-100 for 10, 15, 20, 25 or 30 min at room temperature. The proteolysis reaction was terminated with electrophoresis buffer, and an equal amount of virus lysates were analysed using western blotting.

### Coimmunoprecipitation (Co-IP)

Co-IP was used to investigate the possible interaction between ORF9 and membrane proteins in v-Oka-infected ARPE-19 cells. Briefly, infected ARPE-19 cell lysates were precleared with protein A beads for at least 2 h at room temperature while rotating. The supernatant of precleared cell lysates incubated overnight with mAbs and protein A beads at 4 °C to form the immunoprecipitation mAb-bead complex. The mAb-bound beads were washed, resuspended in electrophoresis buffer and analysed using western blotting.

### Flow cytometry testing

Flow cytometry was used to analyse the membrane location of ORF9. v-Oka-infected ARPE-19 and uninfected ARPE-19 cells were incubated with 8H6-FITC at 37 °C or 4 °C for 30 min. v-Oka-infected ARPE-19 cells incubated with PBS at 4 °C for 30 min were used as the controls. All the samples were analysed using a FACSAria III cell sorter (BD Biosciences, San Jose, California, USA).

## Additional Information

**How to cite this article**: Liu, J. *et al.* Serological Evaluation of Immunity to the Varicella-Zoster Virus Based on a Novel Competitive Enzyme-Linked Immunosorbent Assay. *Sci. Rep.* **6**, 20577; doi: 10.1038/srep20577 (2016).

## Figures and Tables

**Table 1:** Production of recombinant VZV membrane proteins and mAbs.

Protein name	Expression system	Expression segment (aa)	Fusion tag	Expression form	Purify method	mAbs
rgE	Baculovirus	1–537	His tag	Secretion expression	Ni-NTA	70 41
rgB	*E. coli*	23–382	His tag	Inclusion body	Electroelution	5
rgH	*E. coli*	518–804	His tag	Inclusion body	Ni-NTA	3
rgI	*E. coli*	21–274	GST tag	Soluble expression	Glutathione Sepharose 4B	2
rgC	*E. coli*	18–407	GST tag	Soluble expression	Glutathione Sepharose 4B	3
rgK	*E. coli*	22–114	GST tag	Inclusion body	Electroelution	2
rgL	*E. coli*	23–160	His tag	Inclusion body	Ni-NTA	4
rgM	*E. coli*	2–35	GST tag	Soluble expression	Glutathione Sepharose 4B	3
rgN	*E. coli*	21–49	His tag	Soluble expression	Ni-NTA	2
VZV gps					Lentil lectin Sepharose 4B	7
Cell-free virus						9

**Table 2:** Evaluation of human serum using the competitive sandwich ELISA in comparison to the FAMA test.

Competitive sandwich ELISA	FAMA test		
	Positive (FAMA titre ≥1:8)	Negative (FAMA titre <1:8)	Total
Serum from child (group I, 9-month-old to 12-years-old)			
Positive (blocking rate ≥ 50%)	360	1	361
Negative (blocking rate < 50%)	20	439	459
Total	380	440	820
Serum from adults (group II)			
Positive (blocking rate ≥50%)	96	0	96
Negative (blocking rate <50%)	1	3	4
Total	97	3	100

**Table 3:** Performance of the competitive sandwich ELISA for human sera evaluation.

Sensitivity			Specificity			Coincidence		
Group I	Group II	Total	Group I	Group II	Total	Group I	Group II	Total
94.7% (360/380)	98.97% (96/97)	99.77% (456/477)	99.77% (360/361)	100% (96/96)	99.77% (456/457)	97.19% (799/820)	99% (99/100)	97.61% (898/920)
